# The triglyceride-glucose index as a biomarker of diabetic retinopathy: a systematic review and meta-analysis

**DOI:** 10.3389/fmed.2025.1677818

**Published:** 2025-10-27

**Authors:** Adilet Amirashov, Aigul Balmukhanova, Arip Koishybayev, Denis Petrachkov, Dana Koishybayeva, Altynay Balmukhanova, Nadiar M. Mussin, Amin Tamadon

**Affiliations:** ^1^Department of Otorhinolaryngology, Ophthalmology, West Kazakhstan Marat Ospanov Medical University, Aktobe, Kazakhstan; ^2^Department of Surgical Disciplines, Caspian University, Almaty, Kazakhstan; ^3^Department of Oncology, West Kazakhstan Marat Ospanov Medical University, Aktobe, Kazakhstan; ^4^Krasnov Research Institute of Eye Diseases, Moscow, Russia; ^5^Department of Surgical Diseases-2, West Kazakhstan Marat Ospanov Medical University, Aktobe, Kazakhstan; ^6^Department of Policy and Healthcare Management, Al-Farabi Kazakh National University, Almaty, Kazakhstan; ^7^Department of Surgery No. 2, West Kazakhstan Marat Ospanov Medical University, Aktobe, Kazakhstan; ^8^Department of Natural Sciences, West Kazakhstan Marat Ospanov Medical University, Aktobe, Kazakhstan

**Keywords:** triglyceride-glucose index, TyG, TyG index, diabetes mellitus, diabetic retinopathy, meta-analysis

## Abstract

**Background:**

The triglyceride-glucose (TyG) index, a surrogate marker of insulin resistance, has been linked to various diabetic complications. However, its association with diabetic retinopathy (DR) remains inconsistent. We conducted a systematic review and meta-analysis to evaluate the relationship between TyG index levels and the risk of DR.

**Methods:**

We searched PubMed, Scopus, and Web of Science from inception to July 2025 for observational studies reporting the association between TyG index and DR in adults with type 1 or type 2 diabetes. Two reviewers independently screened studies, extracted data, and assessed risk of bias using the Agency for Healthcare Research and Quality (AHRQ) checklist and Newcastle-Ottawa Scale. Pooled odds ratios (ORs) with 95% confidence intervals (CIs) were calculated using a random-effects model. Heterogeneity was evaluated with the I^2^ statistic. Publication bias was assessed via funnel plots and Egger's test. Subgroup and meta-regression analyses were conducted to explore heterogeneity.

**Results:**

Sixteen studies with a total of 33,436 participants were included. The pooled OR for the association between higher TyG index and DR was 1.89 (95% CI: 1.27–2.82) when TyG was treated as a categorical variable, and 1.57 (95% CI: 1.25–1.98) when treated as continuous. Significant heterogeneity was observed (*I*^2^ > 87%). Subgroup analyses revealed stronger associations in studies with smaller sample sizes and higher male proportions. Meta-regression showed that male proportion accounted for 48.71% of the heterogeneity. In categorical analyses, funnel-plot asymmetry and Egger's test indicated small-study effects; after trim-and-fill adjustment the pooled effect attenuated and was no longer significant, suggesting sensitivity to publication bias.

**Conclusions:**

While higher TyG levels correlate with DR—particularly when modeled continuously—the signal is heterogeneity- and bias-sensitive in categorical analyses. Our moderator analyses newly indicate a sex-composition effect, and the current lack of harmonized clinical TyG thresholds limits immediate translation.

## 1 Introduction

Diabetic retinopathy (DR) is a major microvascular complication of diabetes mellitus, characterized by progressive damage to the retinal vasculature, which can ultimately lead to visual impairment and blindness (1, 2). It affects approximately 30% of individuals with diabetes and remains one of the leading causes of vision loss among working-age adults globally (2, 3). The pathogenesis of DR is multifactorial, involving oxidative stress, chronic inflammation, and neurovascular dysfunction, all of which contribute to both microvascular injury and neuronal degeneration in the retina (4–6).

The triglyceride–glucose (TyG) index, a surrogate marker of insulin resistance, was calculated using the following formula: TyG = ln (*fastingtriglycerides*[*mg*/*dL*] × *fastingglucose*[*mg*/*dL*])/2, where ln denotes the natural logarithm (base e). When triglyceride or glucose values were reported in mmol/L, they were converted to mg/dL using standard conversion factors (1 mmol/L = 18 mg/dL for glucose; 1 mmol/L = 88.5 mg/dL for triglycerides) to ensure consistency across studies (7, 8). Originally developed to estimate insulin resistance, the TyG index has been increasingly recognized as a predictor of type 2 diabetes and its vascular complications, including nephropathy, non-alcoholic fatty liver disease, and coronary artery disease (9–13). While most research to date has focused on its association with diabetic nephropathy and cardiovascular outcomes, recent studies have begun to investigate its potential link with DR (14, 15). However, findings across these studies are inconsistent, likely due to heterogeneity in study design, patient populations, and outcome definitions. These discrepancies highlight the need for a comprehensive evaluation of the TyG index's association with DR (4, 16). Accordingly, this systematic review and meta-analysis aims to synthesize the available evidence from observational studies to quantify the strength of this association, identify sources of heterogeneity, and evaluate the TyG index's utility as a predictive biomarker for DR. The findings of this review may inform clinical decision-making by supporting the use of the TyG index as a simple, non-invasive screening tool for early DR detection, particularly in resource-constrained settings.

Despite two prior meta-analyses suggesting a positive TyG–DR association, important questions remain unresolved: (i) how much of the signal persists after explicitly modeling small-study/publication bias; (ii) which study-level factors explain the striking heterogeneity; and (iii) whether heterogeneous and non-standardized TyG cut-offs undermine clinical translation. We therefore prespecified a bias-aware synthesis (trim-and-fill; contour-enhanced funnel inspection) and moderator/meta-regression analyses, and we systematically compiled TyG thresholds used across studies.

## 2 Materials and methods

### 2.1 Search strategy

This systematic review and meta-analysis was conducted in accordance with the PRISMA 2020 guidelines and prospectively registered in PROSPERO (Registration ID: CRD420251110467). A comprehensive literature search was performed in PubMed, Scopus, and Web of Science from inception to July 28, 2025. The search and screening procedures were conducted after protocol registration in PROSPERO (ID: CRD420251110467), and registration preceded data extraction and analysis. We used a combination of MeSH terms and free-text keywords related to the TyG index and DR, with full search details provided in Table 1. To maximize sensitivity, no filters were applied regarding language, publication date, or study design. Additionally, we conducted manual searches of reference lists from relevant articles, reviewed gray literature sources including conference abstracts and dissertations, to identify any eligible unpublished studies.

**Table 1:** Queries used in PubMed, Scopus, and Web of Science databases for a systematic review and meta-analysis of the studies evaluated the triglyceride-glucose index as a biomarker of diabetic retinopathy.

**No**	**Queries**
#1	“triglyceride glucose index” OR “TyG index” OR “triglyceride and glucose index” OR “triglyceride–glucose index” OR “triglyceride/glucose index” OR “triacylglycerol glucose index”
#2	“diabetic retinopathy” OR “diabetic retinopathies” OR “diabetes retinopathy” OR “retinopathy, diabetic”
#3	#1 and #2

The search strategy was developed using the PICO framework: Population (P) included adults with type 1 or type 2 diabetes mellitus; Indicator (I) was the TyG index, Comparator (C) included reference or lower TyG levels, when applicable; Outcome (O) was the presence, severity, or progression of DR, reported as odds ratios (ORs), hazard ratios (HRs), or relative risks (RRs), each with 95% confidence intervals (CIs). Eligible studies included original observational designs (cross-sectional, cohort, or case-control). We excluded studies that did not report TyG-related data or outcome measures for DR, as well as systematic reviews, meta-analyses, case reports, editorials, and duplicate or overlapping datasets. Population (P): adults with diabetes mellitus (type 1 or type 2); Indicator (I): TyG index; Comparator (C): reference or lower TyG levels (where applicable); Outcome (O): presence, severity, or progression of DR. Only original observational studies (cross-sectional, cohort, or case-control).

### 2.2 Selection of studies

**Figure 1:**
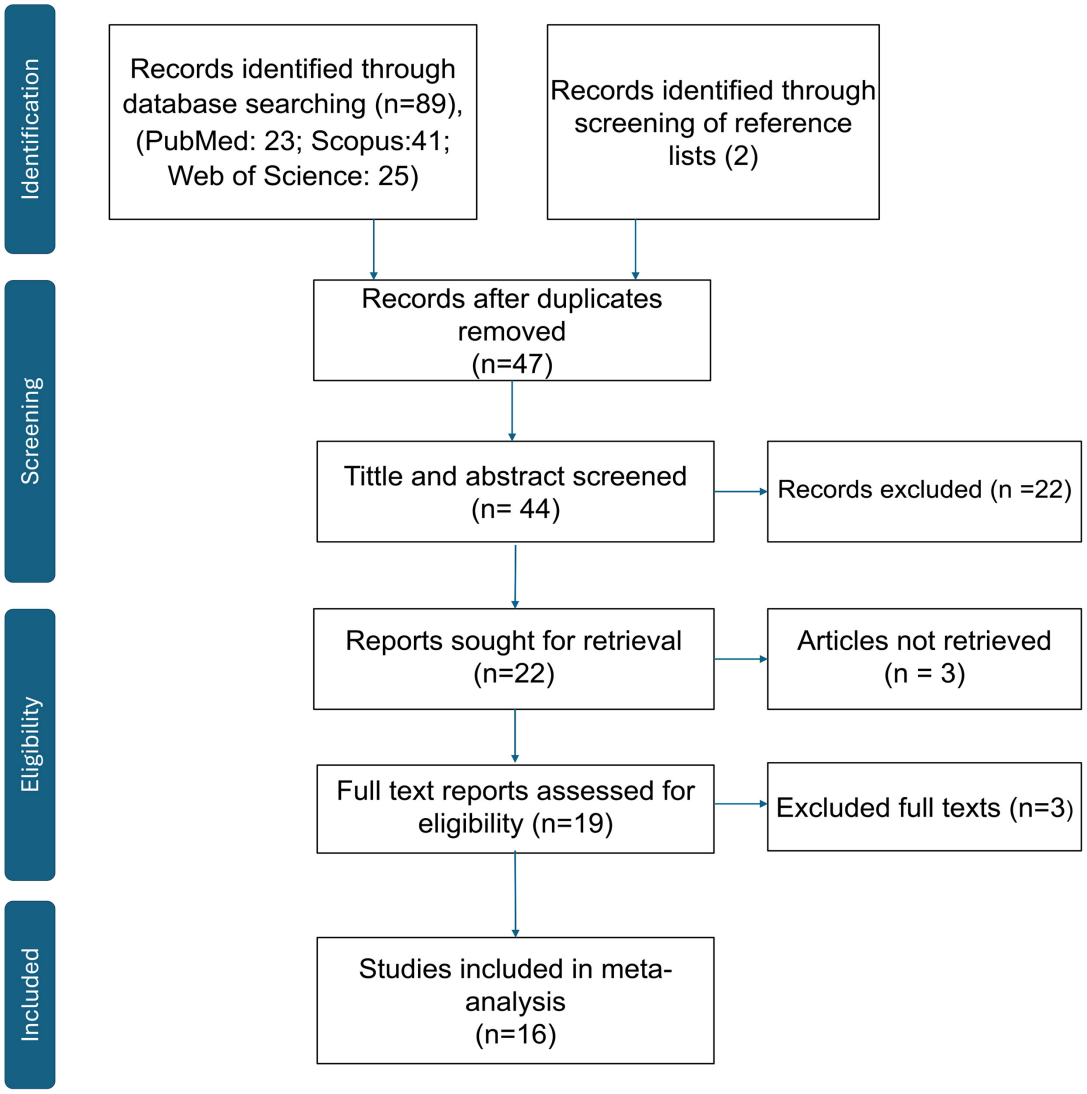
PRISMA 2020 flow diagram. Records identified, screened, assessed for eligibility, and included in the meta-analysis. Records excluded with reasons (n = 27); see Supplementary Table S1 for the full list and reasons.

All retrieved references were imported into EndNote X21 for deduplication. Two independent reviewers screened titles and abstracts, followed by full-text assessments based on predefined inclusion and exclusion criteria. Discrepancies were resolved by consensus or through consultation with a third reviewer. The overall selection process is illustrated in the PRISMA flow diagram (Figure 1), and studies excluded with reasons are presented in Supplementary Table S1.

### 2.3 Data extraction

Data extraction was performed independently by two reviewers (A.A. and A.T.) using a standardized Microsoft Excel spreadsheet. Extracted information included the first author's name, year of publication, country, study design, total sample size, mean age, study design, gender, and effect estimates such as ORs, HRs, or RRs with corresponding 95% CIs. We also recorded the variables included in multivariable models for adjustment. The TyG index was extracted and analyzed both as a categorical variable (quartiles) and as a continuous variable (per 1-unit or 1-standard deviation increase). When multiple effect estimates were reported within a study, we extracted the fully adjusted model to minimize confounding bias. For meta-analytic pooling, odds ratios (ORs), hazard ratios (HRs), and relative risks (RRs) were treated as equivalent measures of association, given the low incidence of diabetic retinopathy in most populations. When necessary, HRs and RRs were considered approximate ORs and combined using the generic inverse-variance method, consistent with established meta-analytic practice. All effect measures were standardized to represent the risk of DR associated with higher TyG index values (either per 1-unit increase or highest vs. lowest category).

### 2.4 Quality assessment

The methodological quality (risk of bias) of each included study was independently assessed by two reviewers (A.A. and A.T.). For cross-sectional studies, we applied the Agency for Healthcare Research and Quality (AHRQ) checklist; for cohort and case-control studies, we used the Newcastle–Ottawa Scale (NOS). Each tool was applied according to its official guidance, and disagreements were resolved by consensus. All disagreements in quality scoring were resolved by discussion and consensus (17).

### 2.5 Statistical analysis

All statistical analyses were conducted using R Studio version 4.5.0 (RStudio, PBC, Boston, MA, USA) with the meta, metafor, and dmetar packages. Pooled effect sizes (ORs, HRs, or RRs) were calculated with 95% CIs using fixed-effects models when heterogeneity was low (*I*^2^ ≤ 50%) and random-effects models when heterogeneity was substantial (*I*^2^ > 50%). Predefined subgroup analyses were conducted by study design (cohort, cross-sectional), sample size, year, and gender. To further explore sources of heterogeneity, meta-regression was performed using covariates such as mean age, sample size, year and gender. Sensitivity analyses were conducted using a leave-one-out method to evaluate the robustness of the pooled estimates. Publication bias was assessed using funnel plot visualization and Egger's regression test, with *p-values* less than 0.10 considered indicative of potential bias (18).

In addition to conventional Egger's test, we (i) inspected contour-enhanced funnel plots to discern whether asymmetry aligned with statistical significance contours, and (ii) conducted Duval and Tweedie trim-and-fill for sensitivity to small-study effects. Predefined meta-regressions evaluated year, sample size, study design, mean age, and male proportion as moderators. We also extracted any reported TyG cut-offs used for categorization and tabulated them (Supplementary Table S2) to appraise threshold variability.

## 3 Results

### 3.1 Characteristics of included studies

A total of 16 observational studies published between 2019 and 2025 were included in the systematic review and meta-analysis, encompassing 33,436 participants with type 2 diabetes mellitus across multiple countries, predominantly from East and South Asia. The included studies comprised 12 cross-sectional studies, 3 cohort studies, and 1 case-control study. Sample sizes varied widely, ranging from 154 to 13,394 participants, with mean participant ages spanning from 53.5 to 64.2 years. The proportion of male participants ranged from 43% to 86%.

The TyG index was analyzed either as a categorical variable or as a continuous variable. Four studies presented TyG solely as a categorical variable, seven studies assessed it exclusively as a continuous variable, and five studies reported TyG in both formats. Most studies adjusted for key covariates including age, sex, diabetes duration, glycemic control (HbA1c), body mass index (BMI), blood pressure, and lipid parameters. However, the degree of covariate adjustment varied across studies, with only a subset including medications, lifestyle factors, or markers of renal function. However, the set of covariates adjusted for varied considerably among studies. Only a subset included lifestyle factors such as smoking, alcohol consumption, or physical activity, while others controlled for renal or hepatic function markers. Several cross-sectional studies provided only minimal adjustment for age and sex. This heterogeneity in model adjustment may contribute to residual confounding and the between-study variability observed in pooled estimates.

Geographically, the majority of studies (*n* = 11) were conducted in China, while the remainder originated from USA, India, Egypt, Iraq, and Singapore. Across studies, DR was diagnosed based on fundus photography or clinical ophthalmologic examination, although specific grading scales were not consistently reported. A detailed summary of study-level characteristics, including country, study design, sample size, age, sex distribution, and adjusted variables, is presented in Table 2. Most studies classified DR as a binary outcome (presence vs. absence), and only a few reported severity grading (e.g., non-proliferative vs. proliferative). However, separate effect estimates by DR stage were not available, preventing stratified quantitative synthesis by disease severity.

**Table 2:** Summary of included studies in a systematic review and meta-analysis of the studies evaluated the triglyceride-glucose index as a biomarker of diabetic retinopathy.

**Authors, year (Reference)**	**Country**	**Type of study**	**Sample size**	**Mean age (years)**	**Male (%)**	**TyG index analysis**	**Variables adjusted**
Chiu et al., 2020 (19)	Taiwan	Retrospective cross-sectional	1,990	64.2 ± 10.6	43%	Q1:Q4	Age, BMI, eGFR, HbA1c, PP, Sex, Statin or fibrate use, TC, WC
Hameed et al., 2019 (14)	Irak	Retrospective cross-sectional	416	NDR: 54.56 ± 9.31 DR: 58.98 ± 7.63	47%	Q1:Q4	Age, BMI, DBP, Duration of diabetes, FBG, HbA1c, HDL, SBP, TC, TG, WC
Kassab et al., 2023 (20)	Egypt	Cross-sectional	500	54.0 ± 8.6	45%	Continuous	Age, Duration of diabetes, Sex HbA1c, uACR
Li et al., 2025 (21)	China	Retrospective cross-sectional	200	–	53%	Continuous	Age, Sex, T2DM duration, Blood pressure, Diabetic duration, HbA1c, CRP, FBG
Li et al., 2022 (15)	China	Cohort	1,153	58.89 ± 8.60	86%	Q1:Q4 Continuous	Age, Alcohol consumption, BMI, Exercise, HbA1c, HDL-C, SBP, Sex, Smoking, Use of hypoglycemic drugs, Use of lipid-lowering drugs
Neelam et al., 2023 (22)	Singapore	Cohort	13,394	NDR: 56.1 ± 10.7 DR: 56.5 ± 9.4	56%	Continuous	BMI, Duration of type 2 diabetes, eGFR, SBP, uACR
Pan et al., 2021 (4)	China	Cohort	4,721	59.56 ± 13.02	53.57%	Continuous	Age, BMI, Sex, Smoking
Pang et al., 2020 (23)	China	Cross-sectional	208	NDR: 53.68 ± 14.39 DR: 54.85 ± 11.37	61%	Continuous	SBP, SUA, Duration of diabetes
Shan et al., 2022 (16)	China	Retrospective cross-sectional	456	53.54 ± 12.13	64%	Continuous	Duration of diabetes, Smoking, Alcohol consumption, Exercise, SBP, TC, HDL-c, LDL-c, eGFR, BMI, HbA1c, Concurrent medications
Shang et al., 2024 (24)	China	Retrospective cross-sectional	154	NDR: 58.29 ± 10.42 DR: 57.31 ± 9.67	54%	Continuous	Sex, Smoking, Alcohol consumption, UA
Srinivasan et al., 2021 (25)	India	Cross-sectional	1,413	56.30 ± 10	53%	Q1:Q4 Continuous	Age, Smoking, Blood pressure
Wan et al., 2025 (26)	China	Retrospective cross-sectional	437	57.10 ± 12.04	57	Q1:Q4 Continuous	Age, BMI, T2DM duration, Sex, SBP, DBP, BUN, Cr, ALB, hsCRP, LP(a), TC, HDL-C, LDL-C, HbA1c
Wang et al., 2022 (27)	China	Cross-sectional	1,061	NDR: 60.07 ± 8.06 DR: 57.63 ± 8.45	82%	Q1:Q4	Age, Sex, Smoking, Course of diabetes, HbA1c, SBP, DBP, BMI, SUA
Yao et al., 2025 (28)	China	Retrospective cross-sectional	398	NDR: 55.29 ± 12.10 DR: 58.43 ± 11.32	55%	Q1:Q4 Continuous	Age, BMI, Blood pressure, T2DM duration, Sex.
Yao et al., 2021 (29)	China	Case-control	2,112	56.08 ± 13.85	58%	Q1:Q4	Age, BMI, Duration of diabetes, HbA1c, Height, HR, PP, SBP, Sex, TC, Use of antidiabetic agents, Weight
Zhou et al., 2023 (30)	USA	Retrospective cross-sectional	888	62.2 ± 12.1	50%	Q1:Q4 Continuous	Age, Education, HDL, Hypertension, LDL, PIR, Race, Sex, TC

TyG, Triglyceride-Glucose Index; DR, Diabetic Retinopathy; NDR, No Diabetic Retinopathy; T2DM, Type 2 Diabetes Mellitus; BMI, Body Mass Index; WC, Waist Circumference; FBG, Fasting Blood Glucose; HbA1c, Glycated Hemoglobin; SBP, Systolic Blood Pressure; DBP, Diastolic Blood Pressure; TC, Total Cholesterol; TG, Triglycerides; HDL/HDL-C, High-Density Lipoprotein (Cholesterol); LDL/LDL-C, Low-Density Lipoprotein (Cholesterol); SUA, Serum Uric Acid; HR, Heart Rate; PP, Pulse Pressure; eGFR, Estimated Glomerular Filtration Rate; uACR, Urine Albumin-to-Creatinine Ratio; hsCRP, High-Sensitivity C-Reactive Protein; LP(a), Lipoprotein (a); BUN, Blood Urea Nitrogen; Cr, Creatinine; ALB, Albumin; PIR, Poverty-Income Ratio; UA, Uric Acid.

### 3.2 Methodological quality and bias risk

The methodological quality of the included observational studies was assessed using validated tools appropriate to study design. For the 12 cross-sectional studies, we employed the AHRQ checklist, which evaluates 11 methodological domains including source of information, inclusion criteria, confounder control, and handling of missing data. Studies achieving scores of ≥7 out of 11 were considered high quality.

Among the cross-sectional studies, four achieved a score of 7 or higher, indicating good methodological rigor, while the remaining studies scored between 4 and 6, reflecting moderate quality. Common limitations among cross-sectional studies included unclear reporting on masking of evaluators, quality assurance procedures, and handling of missing or incomplete data.

For the four cohort and case-control studies, we applied the Newcastle-Ottawa Scale (NOS), which evaluates studies across three domains: selection, comparability, and exposure/outcome assessment, with a maximum score of 9. All cohort and case-control studies were rated as high quality, with scores ranging from 8 to 9, demonstrating strong methodological integrity. These studies generally showed adequate selection of study groups, control of confounding, and consistent ascertainment of exposure.

Overall, the included studies were of moderate to high quality. However, potential sources of bias remained due to heterogeneity in covariate adjustment, incomplete methodological reporting, and variability in diagnostic criteria for DR. These concerns were addressed through sensitivity analyses, subgroup analyses, and meta-regression to identify and account for potential sources of heterogeneity. Detailed quality assessment scores are presented in Table 3 (AHRQ) and Table 4 (NOS).

**Table 3:** Risk-of-bias assessment of cross-sectional studies using the Agency for Healthcare Research and Quality (AHRQ) checklist.

**Authors, year (Reference)**	**1**	**2**	**3**	**4**	**5**	**6**	**7**	**8**	**9**	**10**	**11**	**Total**
Chiu et al., 2020 (19)	Y	Y	Y	N	N	Y	Y	Y	N	N	N	6/11
Hameed et al., 2019 (14)	Y	Y	Y	Y	N	Y	Y	Y	U	U	N	7/11
Kassab et al., 2023(20)	Y	Y	Y	U	U	Y	Y	Y	N	N	N	6/11
Li et al., 2025 (21)	Y	Y	Y	U	N	N	Y	Y	U	U	N	6/11
Pang et al., 2020 (23)	Y	Y	Y	Y	N	Y	N	Y	U	U	N	6/11
Shan et al., 2022 (16)	Y	Y	Y	Y	U	Y	Y	Y	N	N	N	7/11
Shang et al., 2024 (24)	Y	Y	Y	U	N	N	Y	Y	U	Y	N	6/11
Srinivasan et al., 2021 (25)	Y	Y	N	N	N	Y	U	Y	U	N	N	4/11
Wan et al., 2025 (26)	Y	Y	Y	U	N	U	Y	Y	U	Y	N	6/11
Wang et al., 2022 (27)	Y	Y	Y	U	N	Y	Y	Y	U	Y	N	7/11
Yao et al., 2025 (28)	Y	Y	Y	U	N	N	Y	Y	U	Y	N	6/11
Zhou et al., 2023 (30)	Y	Y	Y	U	N	Y	Y	Y	U	Y	N	7/11

1 Define source of information (survey, record review).

2 List inclusion and exclusion criteria for exposed and unexposed subjects (cases and controls) or refer to previous publications.

3 Indicate time period used for identifying patients.

4 Indicate whether or not subjects were consecutive if not population-based.

5 Indicate if evaluators of subjective components of study were masked to other aspects of the status of the participants.

6 Describe any assessments undertaken for quality assurance purposes.

7 Explain any patient exclusions from analysis.

8 Describe how confounding was assessed and/or controlled.

9 If applicable, explain how missing data were handled in the analysis.

10 Summarize patient response rates and completeness of data collection.

11 Clarify what follow-up, if any, was expected and the percentage of patients for which incomplete data or follow-up was obtained.

**Table 4:** Risk-of-bias assessment of cohort and case–control studies using the Newcastle–Ottawa Scale (NOS).

**Authors, year (Reference)**	**Selection**				**Comparability**		**Exposure**			**Total**
	**Case definition adequate**	**Representativeness of the cases**	**Selection of controls**	**Definition of controls**	**Comparability of cases and controls**	**Control for additional confounders**	**Ascertainment of exposure**	**Same method of ascertainment for groups**	**Non-response rate**	
Li et al., 2022 (15)	⋆	⋆	⋆	✰	⋆	⋆	⋆	⋆	⋆	8/9
Neelam et al., 2023 (22)	⋆	⋆	⋆	⋆	⋆	⋆	⋆	⋆	✰	8/9
Pan et al., 2021 (4)	⋆	⋆	⋆	⋆	⋆	⋆	⋆	⋆	✰	8/9
Yao et al., 2021 (29)	⋆	⋆	⋆	⋆	⋆	⋆	⋆	⋆	⋆	9/9

✰-0 point, ⋆-1 point.

### 3.3 TyG as a categorical variable

**Figure 2:**
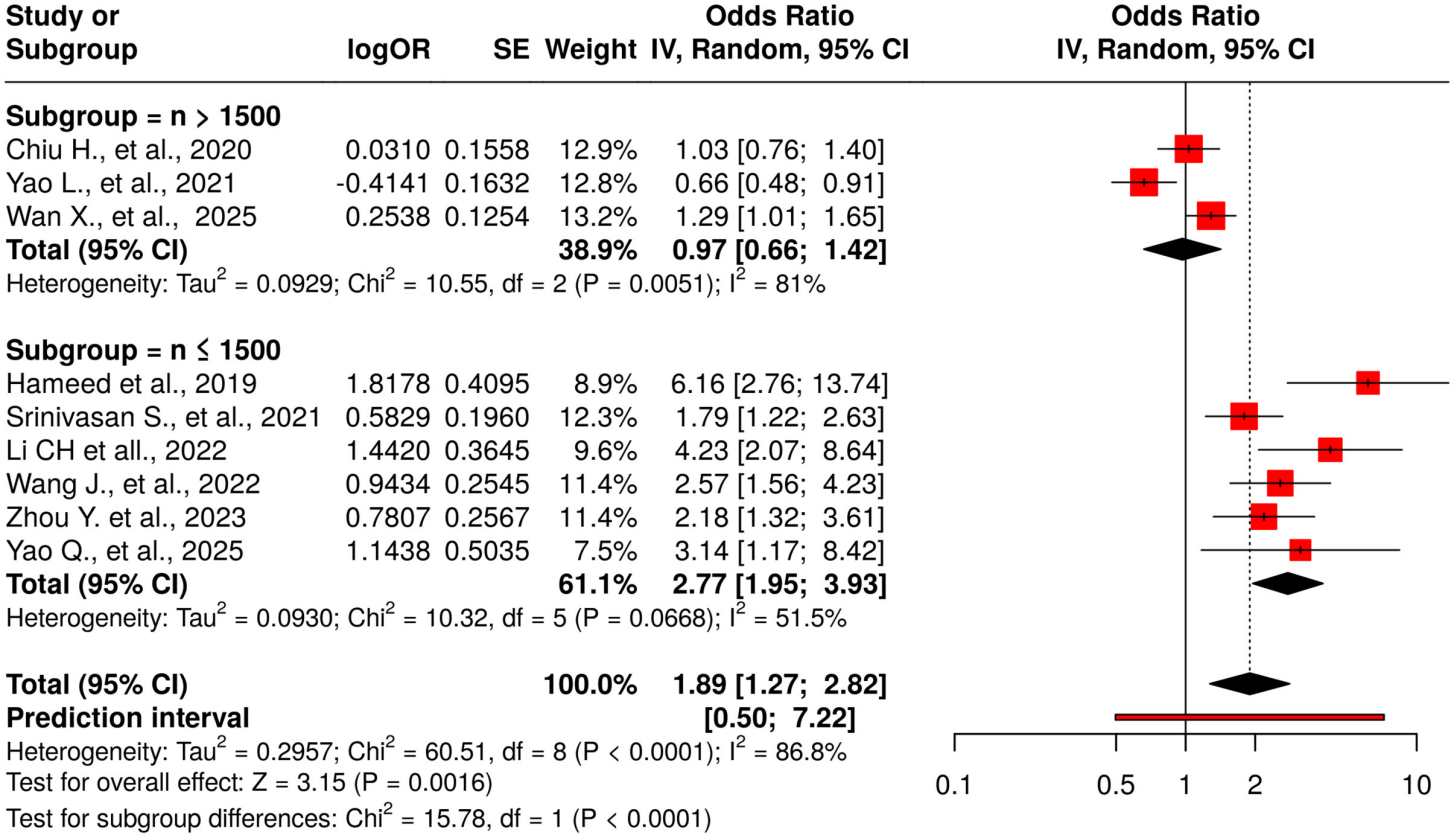
Forest plot for TyG as a categorical exposure (highest vs. lowest category). Random-effects model (DerSimonian–Laird): pooled OR = 1.89 (95% CI, 1.27–2.82); I^2^ = 87%, τ^2^ = 0.45. Funnel-plot asymmetry consistent with small-study effects (Egger's p = 0.016). After Duval & Tweedie trim-and-fill (4 imputed studies), the pooled effect attenuated to non-significance (OR = 0.93, 95% CI, 0.54–1.61).

**Figure 3:**
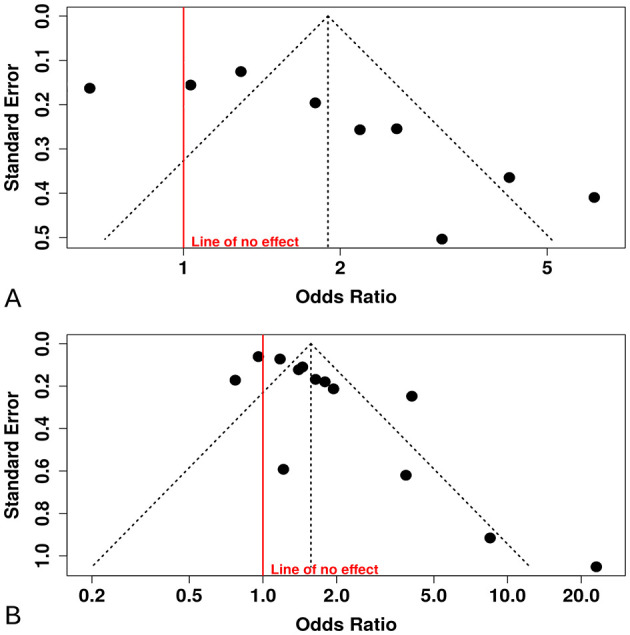
Funnel plots with Egger's tests. **(A)** TyG categorical (highest vs. lowest): Egger's intercept = 5.09 (95% CI, 1.94–8.2), *p* = 0.016; Duval & Tweedie trim-and-fill imputed 4 studies; adjusted pooled effect became non-significant. **(B)** TyG continuous (per 1-unit increase): Egger's intercept = 2.87 (95% CI, 1.17–4.57), *p* = 0.007.

Nine studies were included in the meta-analysis evaluating the TyG index as a categorical variable Figure 2. The initial random-effects model revealed a pooled OR of 1.89 (95% CI: 1.27–2.82; p < 0.03), indicating a significant association between elevated TyG index and DR. However, significant heterogeneity was observed (I^2^ = 87%, p < 0.01). Significant funnel-plot asymmetry was detected (Egger's p = 0.016), confirming potential publication bias (Figure 3A). Applying the Duval and Tweedie trim-and-fill procedure, four hypothetical studies were imputed to restore symmetry. The bias-adjusted pooled odds ratio decreased to 0.93 (95% CI 0.54–1.61) and was no longer statistically significant, indicating that the initial positive association was not robust after accounting for small-study effects. This suggests that the apparent relationship between categorical TyG levels and DR may be partly influenced by selective publication of positive results.

To address potential bias, trim-and-fill analysis was performed, imputing 4 hypothetical studies to symmetrize the funnel plot. After adjustment, the pooled OR attenuated to 0.93 (95% CI: 0.54–1.61; *p* = 0.35), with no significant overall effect. Heterogeneity remained high (*I*^2^ = 89%, Tau^2^ = 0.45; *p* < 0.0001), suggesting persistent variability beyond sampling error.

Subgroup analysis based on study sample size revealed substantial differences. For studies with a sample size greater than 1,500 participants (*n* = 3), the pooled OR was 0.97 (95% CI: 0.66–1.42), indicating no significant association, albeit with high heterogeneity (*I*^2^ = 81%; *p* = 0.005). In contrast, studies with a sample size of 1,500 or fewer participants (*n* = 6) demonstrated a significant positive association (pooled OR: 2.77; 95% CI: 1.95–3.93), with moderate heterogeneity (*I*^2^ = 51.5%; *p* = 0.066). The difference between these subgroups was statistically significant (*p* < 0.0001), highlighting sample size as a potential source of heterogeneity.

Sensitivity analysis using the leave-one-out method confirmed the robustness of the overall initial association. The pooled OR remained statistically significant across all iterations (range: 1.67–2.16), with the lower limits of the 95% CIs consistently above 1.0 (range: 1.143–1.491), confirming the stability of the observed positive relationship. However, heterogeneity remained consistently high (*I*^2^ = 80.5–89%, *p* < 0.01 in all scenarios), underscoring persistent variability not attributable to any single study (Supplementary Table S3).

Given the observed funnel asymmetry, trim-and-fill imputed four studies and attenuated the pooled OR to non-significance, indicating the categorical association is sensitive to small-study/publication bias.

### 3.4 TyG as a continuous variable

**Figure 4:**
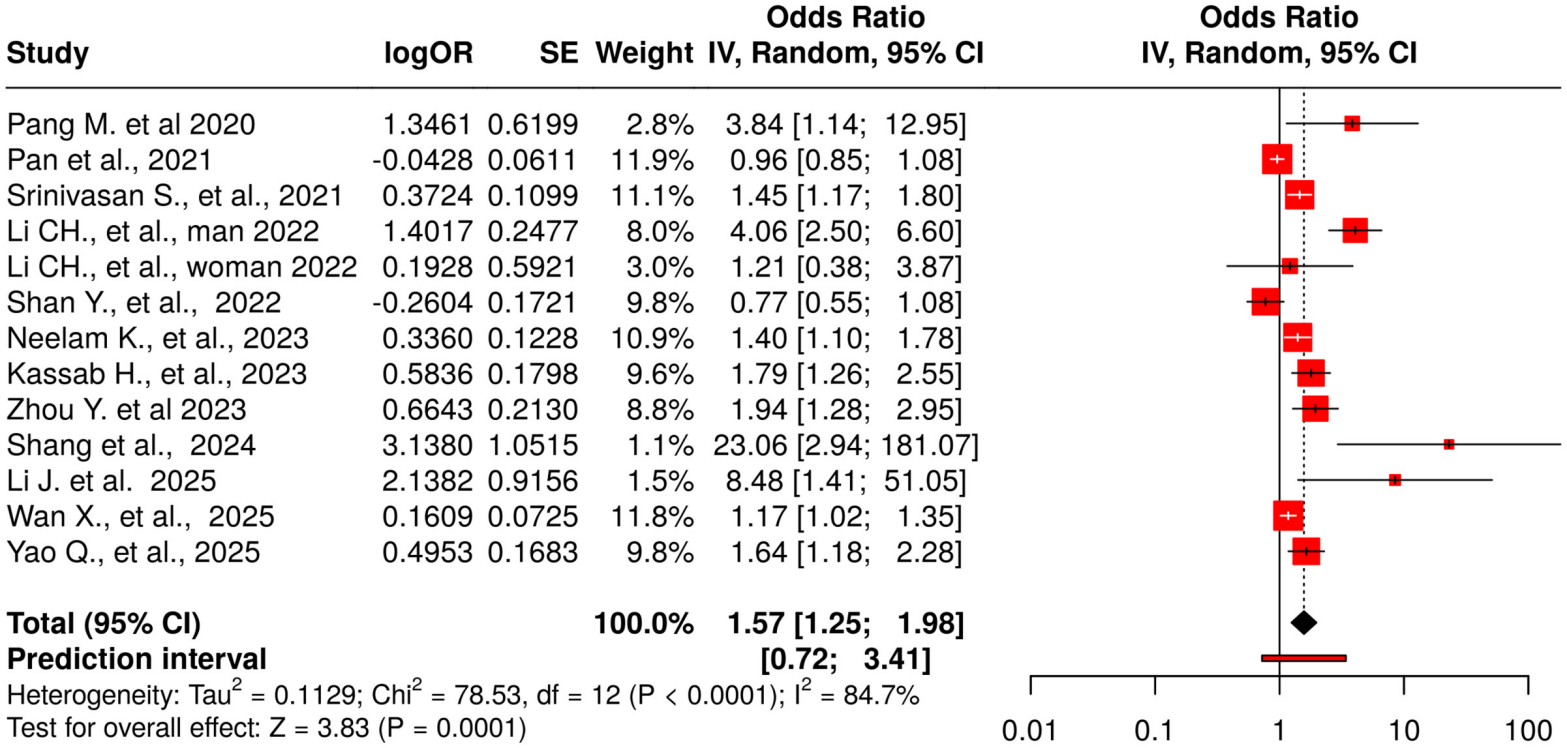
Forest plot for TyG as a continuous exposure (per 1-unit increase in TyG). Random-effects model (REML): pooled OR = 1.57 (95% CI, 1.25–1.98); I^2^ = 85% (report exact), τ^2^ = 0.218 (report exact). Egger's p = 0.007. Meta-regression identified male proportion as a significant moderator (~49% of between-study heterogeneity explained).

Thirteen studies reported the association between the TyG index (as a continuous variable) and DR Figure 4. The pooled analysis demonstrated a statistically significant positive association (OR = 1.57, 95% CI: 1.25–1.98, *p* < 0.05), although substantial heterogeneity was observed among the included studies (*I*^2^ > 87%). The funnel plot suggested potential publication bias, which was confirmed by Egger's test (intercept: 2.87, 95% CI: 1.17–4.57, *t* = 3.307, *p* = 0.007), indicating that smaller studies tended to report stronger effects Figure 3B.

Meta-regression was performed to investigate possible sources of heterogeneity. No significant trend was found with the year of publication (coefficient = 0.0488, *p* = 0.6591, *R*^2^ = 0%), continuous sample size (coefficient = 0, *p* = 0.3179, *R*^2^ = 0%), or study design (*p* = 0.9418, *R*^2^ = 0%), suggesting these variables did not contribute meaningfully to the variability. However, the proportion of male participants significantly explained nearly half of the heterogeneity (coefficient = 0.0210, *p* = 0.0209, *R*^2^ = 49.56%), indicating stronger associations between TyG and DR in studies with a higher percentage of male participants. Additionally, studies were categorized based on sample size (>1,000 vs. ≤ 1,000), revealing a non-significant trend (coefficient = −0.5089, *p* = 0.0859, *R*^2^ = 11.13%), suggesting larger effect sizes in smaller studies. Despite these analyses, substantial residual heterogeneity persisted, indicating the presence of unexplained variability.

To further assess robustness, a leave-one-out sensitivity analysis was conducted. The original random-effects model yielded an OR of 1.6606 (95% CI: 1.1200–2.4620, *p* = 0.0159) with substantial heterogeneity (*I*^2^ = 84.7%, tau^2^ = 0.2176). The leave-one-out analysis demonstrated that exclusion of individual studies did not eliminate the statistically significant positive association, with ORs ranging from 1.4294 to 1.7706. The largest reduction in OR (to 1.4294) occurred after excluding Li et al. (11) (male subgroup), indicating this study had the most substantial influence Supplementary Table S4. Shang et al. (24) and Pang et al. (23) also notably influenced the overall pooled effect. Nevertheless, no single study fully accounted for the heterogeneity, which remained high (*I*^2^ > 79%) across all iterations. Exclusion of Li et al. (11) (female subgroup) resulted in the highest heterogeneity (*I*^2^ = 86.0%), whereas removal of Li et al. (11) (male subgroup) slightly reduced it (*I*^2^ = 79.6%).

In summary, the positive association between continuous TyG index and DR remained robust across multiple sensitivity checks, despite evidence of substantial heterogeneity and potential publication bias. Notably influential studies with exceptionally high effect estimates—such as Li et al. (11) (OR = 4.07) and Shang et al. (24) (OR = 23.057)—appeared to pull the pooled estimate upward. These findings underscore both the consistency and complexity of the relationship between the TyG index and DR, highlighting potential differences across populations and subgroups.

Meta-regression identified male proportion as a significant moderator (=49% of explained heterogeneity), indicating stronger TyG–DR associations in studies with higher male representation. Other covariates (year, design, sample size as continuous) did not account for heterogeneity.

### 3.5 Study-level TyG thresholds used for categorization

Across studies that categorized TyG, operational cut-offs varied widely (Supplementary Table S2). Most studies used quartile or quintile splits of cohort-specific TyG distributions, whereas a few applied receiver-operating-characteristic (ROC)-derived thresholds. No study validated its chosen cut-off in an external cohort. The lack of a standardized or clinically justified TyG threshold likely contributes to between-study heterogeneity and limits the biomarker's immediate applicability in clinical settings.

## 4 Discussion

This systematic review and meta-analysis provides comprehensive evidence on the association between the TyG index and DR. The pooled analysis revealed a statistically significant positive relationship between elevated TyG index and DR, with an OR of 1.89 (95% CI: 1.27–2.82) when TyG was analyzed as a categorical variable, and an OR of 1.57 (95% CI: 1.25–1.98) when assessed as a continuous variable. These findings suggest that the TyG index, a surrogate marker for insulin resistance, may serve as a clinically relevant biomarker for DR risk stratification.

However, the significant Egger's test and subsequent loss of statistical significance after trim-and-fill adjustment underscore the potential influence of publication bias in categorical analyses. Consequently, the pooled estimate should be interpreted with caution. The bias-adjusted results suggest that earlier reports may have overestimated the strength of the association, while the continuous-variable analysis—which remained significant across sensitivity tests—likely offers a more reliable estimate of the true relationship.

This review also adds methodological depth by (i) conducting a comprehensive re-appraisal that accounts for publication bias, showing that categorical associations lose significance after bias correction, and (ii) identifying a new moderator—male proportion—that explains approximately 49 % of between-study heterogeneity in continuous-TyG models. These findings, summarized in Supplementary Table S5, refine current understanding of when and in whom TyG best reflects DR risk.

Our results are consistent with previous meta-analyses by Yu et al. (31) and Zhou et al. (30), which also demonstrated significant associations between higher TyG index levels and increased DR risk (32). However, our study extends the existing literature by including a larger pooled sample (33,436 participants across 16 studies) and performing extensive subgroup and sensitivity analyses to identify potential sources of heterogeneity.

A major source of heterogeneity relates to differences in study design, covariate adjustment, and population characteristics. The effect appeared stronger in studies with smaller samples and higher male representation. The latter may reflect sex-specific differences in lipid metabolism, insulin resistance, or vascular vulnerability. Variation in covariate adjustment also likely contributed: while most studies controlled for age, sex, diabetes duration, HbA1c, BMI, and blood pressure, only some accounted for lifestyle factors, medication use, or renal function. The absence of standardized adjustment strategies may have led to residual confounding. Future studies should apply harmonized, comprehensive adjustment models that integrate metabolic, lifestyle, and treatment variables.

The lack of a standardized TyG threshold further limits translation into clinical practice. Reported cut-offs varied widely by cohort and analytic method, and no study validated its threshold externally (Supplementary Table S4). Until prospective, multi-ethnic cohorts establish and validate clinically meaningful cut-points—ideally using ROC or decision-curve analyses—the TyG index should be viewed as a complementary risk marker rather than a stand-alone screening tool.

Geographic and ethnic concentration is another important limitation. Of the 16 studies analyzed, 15 were conducted in East or South Asian populations, mainly in China. This restricts generalizability to non-Asian populations, where differences in genetics, diet, and healthcare systems may modify the TyG–DR relationship. Validation in diverse multi-ethnic cohorts is therefore warranted.

Furthermore, most studies classified DR as a binary outcome rather than by severity stage. As a result, we could not determine whether the TyG index is more strongly associated with early (non-proliferative) or advanced (proliferative) DR. Standardized grading using systems such as the ETDRS classification and provision of stage-specific estimates are needed to clarify the clinical utility of TyG for disease progression monitoring.

From a clinical perspective, the TyG index could be integrated into existing DR screening pathways. Because it relies only on fasting glucose and triglyceride levels—parameters already measured routinely in diabetes care—it offers a low-cost, non-invasive biomarker that could be automatically calculated within electronic health-record systems. Incorporating TyG into risk-stratification algorithms may help identify individuals at higher risk who would benefit from earlier or more frequent retinal evaluation, especially in resource-limited settings. Future research should focus on validating population-specific TyG thresholds, developing predictive models that combine TyG with established risk factors (e.g., HbA1c, diabetes duration, blood pressure, renal function), and assessing the cost-effectiveness of implementation within national screening programs.

### 4.1 Limitations

Several limitations should be acknowledged. First, the predominance of cross-sectional designs limits causal inference and precludes assessing whether TyG predicts DR onset or progression. Second, substantial between-study heterogeneity persisted despite subgroup and meta-regression analyses, so pooled estimates should be interpreted cautiously. Third, publication-bias diagnostics indicated small-study effects in the categorical model, and after Duval–Tweedie trim-and-fill the pooled association became non-significant, suggesting possible overestimation in unadjusted results. Fourth, stage-specific evidence was insufficient: most studies reported DR as a binary outcome, preventing severity-stratified meta-analysis (e.g., NPDR vs. PDR) and limiting analyses by diabetes duration, glycemic control, BMI, and lipid profiles. Fifth, covariate adjustment varied widely; several studies lacked key lifestyle or biochemical controls, raising the possibility of residual confounding. Sixth, there is no standardized or externally validated clinical TyG cut-off, which restricts immediate use in screening or triage. Seventh, the evidence base is geographically concentrated (15/16 studies from Asian populations, predominantly China), limiting generalizability to other ethnic and geographic groups given known differences in insulin sensitivity, lipid metabolism, and microvascular susceptibility (33–35).

### 4.2 Future directions

Future work should (i) undertake sex-stratified, multiethnic prospective cohorts to validate the moderator signal we identified; (ii) perform Mendelian randomization on triglyceride and fasting-glucose instruments to probe causality for microvascular retinal outcomes; (iii) develop externally validated ML risk models that integrate TyG with age, diabetes duration, HbA1c, BP, BMI, and renal markers, reporting decision-curve analysis; and (iv) standardize and externally validate TyG cut-offs using pre-registered protocols.

Addressing these research priorities will be essential for confirming the clinical utility of the TyG index as a non-invasive, cost-effective biomarker for early detection and risk assessment of Dr in diverse populations.

## 5 Conclusions

This systematic review and meta-analysis demonstrates a significant association between the TyG index and DR, supporting its potential as a biomarker for DR risk stratification. However, the observed heterogeneity, publication bias, and geographical limitations (primarily Asian populations) highlight the need for further validation in diverse cohorts and standardized methodologies. Future research should focus on prospective studies, mechanistic insights, and clinical utility to establish the TyG index as a reliable tool for early DR detection and management.

## Data Availability

The raw data supporting the conclusions of this article will be made available by the authors, without undue reservation.
