# Supplementary material for: The triglyceride-glucose index as a biomarker of diabetic retinopathy: a systematic review and meta-analysis
Source: Front Med (Lausanne). 2025 Oct 27;12:1677818. doi: 10.3389/fmed.2025.1677818 (PMC12597932; doi:10.3389/fmed.2025.1677818)
Supplement: Supplementary file 1 [file Table_1.docx]

Table S1. Excluded studies list

| Study ID | Reason of exclusion |
| --- | --- |
| Cao et al 2025 (1) | Articles not retrieved |
| Çatak et al 2025 (2) | Not enugh data |
| Chen et al 2022(3) | Not relevant to the topic |
| Chen et al 2025 (4) | Not relevant to the topic |
| Han et al 2024 (5) | Not relevant to the topic |
| Huang et al 2025(6) | Not relevant to the topic |
| Jiao et al 2023(7) | Articles not retrieved |
| Jin et al 2025 (8) | Not relevant to the topic |
| Kwiendacz et al 2025 (9) | Not relevant to the topic |
| Li et al 2021(10) | Not relevant to the topic |
| Li et al 2024 (11) | Not relevant to the topic |
| Lian et al 2024 (12) | The TyG index is not mentioned |
| Liu et al 2023 (13) | Not relevant to the topic |
| Liu et al 2024 (14) | Not enugh data |
| Nayak et al 2024 (15) | This is a meta-analysis that includes more than just retinopathy |
| Rauscher et al 2024 (16) | Not enugh data |
| Sánchez-Pozos et al 2021(17) | Not relevant to the topic |
| Seufert et al 2024 (18) | Not relevant to the topic |
| Tian et al 2022 (19) | Not relevant to the topic |
| Tong et al 2022 (20) | Not relevant to the topic |
| Wang and Chen 2025 (21) | Diabetic retinopathy is not mentioned |
| Wang et al 2024 (22) | the article discusses TyG-WHR |
| Xiao et al., 2022 | Articles not retrieved |
| Xu et al 2024 (23) | The TyG index is not mentioned |
| Yang et al 2024 (24) | Not relevant to the topic |
| Yasir et al 2022 (25) | Not relevant to the topic |
| Yu et al 2023 (26) | The article is a meta-analysis |
| Zhou et al 2023(27) | The article is a meta-analysis |

Table S2. Reported categorical TyG thresholds used across included studies

| Study (year) | TyG categorization | Exact thresholds / quantiles | Derivation method (quartiles / ROC / other) | DR grading used |
| --- | --- | --- | --- | --- |
| Hameed et al., (28) (Iraq) | Quartiles (Q1–Q4) | Cut-points not reported | Quartile distribution of TyG | Fundus examination; grading not specified |
| Chiu et al., (29) (Taiwan) | Quartiles (Q1–Q4) | Not reported | Quartiles | Fundus photography; DR definition per ADA guidelines |
| Pang et al., (30) (China) | Continuous only – no categorical threshold | – | – | Ophthalmologic exam; NR |
| Srinivasan et al., (31) (India) | Quartiles (Q1–Q4) | Not reported | Quartiles | Standard DR grading (ETDRS) |
| Yao et al., (32) (China) | Quartiles (Q1–Q4) | Not reported | Quartiles | Hospital-based ophthalmologic grading |
| Pan et al., (33) (China) | Continuous only | – | – | Fundus exam; NR |
| Wang et al., (34) (China) | Quartiles (Q1–Q4) | Not reported | Quartiles | Fundus examination (not graded) |
| Li et al., (35) (China) | Quartiles and continuous | Quartiles; cut-points NR | Quartiles / ROC (combined) | Fundus photography; DR graded per Chinese Diabetes Society |
| Shan et al., (36) (China) | Continuous only | – | – | Ophthalmologic exam; NR |
| Zhou et al., (27) (USA) | Quartiles (Q1–Q4) | Q1 < 8.31; Q2 8.31–8.57; Q3 8.57–8.86; Q4 > 8.86 (approx., derived from NHANES) | Quartiles | Retinopathy defined by ETDRS grading of fundus images |
| Kassab et al., (37) (Egypt) | Continuous only | – | – | Fundus exam; DR defined as NPDR or PDR |
| Neelam et al., (38) (Singapore) | Continuous only | – | – | Graded per international ETDRS system |
| Shang et al., (39) (China) | Continuous only | – | – | DR diagnosis by fundus photography; NR |
| Wan et al., (40) (China) | Quartiles (Q1–Q4) | Q1 < 8.40; Q2 8.40–8.64; Q3 8.64–8.89; Q4 > 8.89 | Quartiles | Fundus photography; DR classification NR |
| Li et al., (41) (China) | Continuous only | – | – | Fundus photography; DR defined by ophthalmologist |
| Yao et al., (42) (China) | Quartiles (Q1–Q4) and continuous | Cut-points not reported | Quartiles | Hospital-based fundus examination |

Table S3. Leave out test for TyG as a continuous variable

| Study ID | OR | 95% CI | I^2^ | p-value | Egger's test p-value |
| --- | --- | --- | --- | --- | --- |
| Kassab et al 2023 (37) | 1.68 | 1.19 – 2.44 | 85 | 0.002 | 0.01 |
| Li et al 2022 (35) man | 1.43 | 1.14 – 1.79 | 79.5 | 0.001 | 0.012 |
| Li et al 2022 (35) woman | 1.70 | 1.23 - 2.34 | 86 | 0.001 | 0.004 |
| Li et al 2025 (41) | 1.59 | 1.10-2.12 | 85 | 0.001 | 0.01 |
| Neelam et al 2023 (38) | 1.73 | 1.22 - 2.44 | 85 | 0.001 | 0.031 |
| Pan et al 2021 (33) | 1.77 | 1.29 - 2.43 | 80 | 0.0004 | 0.027 |
| Pang et al 2020 (30) | 1.60 | 1.18 - 2.15 | 85 | 0.002 | 0.013 |
| Shan et al 2022 (36) | 1.76 | 1.32 - 2.36 | 84 | 0.0001 | 0.001 |
| Shang et al 2024 (39) | 1.57 | 1.18-2.07 | 84 | 0.001 | 0.01 |
| Srinivasan et al 2021 (31) | 1.72 | 1.22 - 2.44 | 85 | 0.002 | 0.009 |
| Wan et al 2025 (40) | 1.75 | 1.25-2.46 | 85 | 0.001 | 0.009 |
| Yao et al 2025 (42) | 1.70 | 1.20-2.39 | 85 | 0.002 | 0.01 |
| Zhou et al 2023 (43) | 1.66 | 1.19 - 2.32 | 85 | 0.003 | 0.01 |

Table S4. Leave out test for TyG as a categorical variable

| Study ID | OR | 95% CI | I^2^ | p-value | Egger's test p-value |
| --- | --- | --- | --- | --- | --- |
| Chiu et al 2020 (29) | 2.10 | 1.323 - 3.329 | 87.5 | 0.001 | 0.03 |
| Hameed et al 2019 (28) | 1.667 | 1.143 - 2.432 | 89 | 0.008 | 0.053 |
| Li et al 2022 (35) | 1.725 | 1.161 - 2.563 | 86.2 | 0.00697 | 0.044 |
| Srinivasan et al 2021 (31) | 1.93 | 1.229 - 3.034 | 88 | 0.004 | 0.02 |
| Wan et al 2025 (40) | 2.056 | 1.251 - 3.38 | 88.4 | 0.00445 | 0.009 |
| Wang et al 2022 (34) | 1.821 | 1.195 - 2.775 | 87.1 | 0.00532 | 0.031 |
| Yao et al 2021 (32) | 2.16 | 1.491 - 3.125 | 80.5 | 5e-05 | 0.003 |
| Yao et al 2025 (42) | 1.818 | 1.204 - 2.746 | 87.9 | 0.0045 | 0.019 |
| Zhou et al 2023 (43) | 1.868 | 1.213 - 2.877 | 87.7 | 0.00457 | 0.031 |

Table S5. Prior meta-analyses vs. this study

| Feature | Zhou et al. (27) | Yu and Li (26) | The current study (Amirashov et al., 2025) |
| --- | --- | --- | --- |
| Search end date | mid-2023 | 2024 | July 2025 |
| N studies / participants | ~8–12 / ~? | ~10–14 / ~? | 16 / 33,436 |
| Regions represented | Predominantly Asia | Predominantly Asia | Asia + USA, Egypt, Iraq, Singapore |
| Categorical TyG bias adjustment (trim-and-fill) | Not reported | Not reported | Reported; effect → nonsignificant |
| Moderator analyses | Limited | Limited | Male proportion (≈49% heterogeneity) |
| Threshold synthesis | Not emphasized | Not emphasized | Compiled (Table SNEW-2) |
| Conclusion tone | Positive association | Positive association | Positive for continuous TyG; categorical signal bias-sensitive |

**References**

1. Cao BF, Liu K, Chen HW, Xu ZY, Wang SA, Zhang CY, et al. Impact of baseline and trajectory of the cardiometabolic indices on incident microvascular complications in patients with type 2 diabetes. Atherosclerosis. 2025;407:120407. doi:10.1016/j.atherosclerosis.2025.120407

2. Catak M, Konuk SG, Hepsen S. The cholesterol-HDL-glucose (CHG) index and traditional adiposity markers in predicting diabetic retinopathy and nephropathy. J Diabetes Investig. 2025;16:1487-94. doi:10.1111/jdi.70086

3. Chen S, Mei Q, Guo L, Yang X, Luo W, Qu X, et al. Association between triglyceride-glucose index and atrial fibrillation: A retrospective observational study. Front Endocrinol (Lausanne). 2022;13:1047927. doi:10.3389/fendo.2022.1047927

4. Chen Y, Zhao J, Sun Y, Yang Z, Yang C, Zhu D. Association of the triglyceride glucose index with sudden cardiac death in the patients with diabetic foot ulcer. Diabetes Res Clin Pract. 2025;223:112143. doi:10.1016/j.diabres.2025.112143

5. Han F, Xu C, Hangfu X, Liu Y, Zhang Y, Sun B, et al. Circulating glutamine/glutamate ratio is closely associated with type 2 diabetes and its associated complications. Front Endocrinol (Lausanne). 2024;15:1422674. doi:10.3389/fendo.2024.1422674

6. Huang Z, Liu S, Chen C, Zhang K, Du Y, Zhu X. Optimizing serum 25(OH)D levels to mitigate the risk of age-related ocular diseases: insights from a large-scale prospective cohort study. Nutr J. 2025;24(1):88. doi:10.1186/s12937-025-01156-y

7. Jiao C, Hou C, Li R. Correlation between TyG index and non-proliferative retinopathy in type 2 diabetes mellitus. Chin J Clin Res. 2023;36(5):656-60. doi:10.13429/j.cnki.cjcr.2023.05.004

8. Jin Q, Huang J, Gao L, Zhu J. Association between triglyceride-glucose index and cataract among outpatient US adults. Front Med (Lausanne). 2025;12:1523711. doi:10.3389/fmed.2025.1523711

9. Kwiendacz H, Huang B, Chen Y, Janota O, Irlik K, Liu Y, et al. Predicting major adverse cardiac events in diabetes and chronic kidney disease: a machine learning study from the Silesia Diabetes-Heart Project. Cardiovasc Diabetol. 2025;24(1):76. doi:10.1186/s12933-025-02615-w

10. Li Y, Lin X. Expression of adipocytokines in the serum of patients with diabetic retinopathy and its factors. Recent Adv Ophthalmol. 2021;41(1):57-61. doi:10.13389/j.cnki.rao.2021.0012

11. Li X, Wang Y. Associations of the TyG index with albuminuria and chronic kidney disease in patients with type 2 diabetes. PLoS ONE. 2024;19(10):e0312374. doi:10.1371/journal.pone.0312374

12. Lian XN, Zhu MM. Factors related to type 2 diabetic retinopathy and their clinical application value. Front Endocrinol (Lausanne). 2024;15:1484197. doi:10.3389/fendo.2024.1484197

13. Liu ZR, Bao T, Xue GJ, Xu QY, Gao YX, Zhang M. Correlation between diabetic retinopathy and Helicobacter pylori infection: a cross-sectional retrospective study. Int J Ophthalmol. 2023;16(8):1260-7. doi:10.18240/ijo.2023.08.11

14. Ran L, Han Y, Zhaohu H, Hailin S. Correlation Between Triglyceride-Glucose Index and Microvascular Complications in Patients With Early- Onset of Type 2 Diabetes Mellitus. Endocrinol Diabetes Metab. 2025;8(2):e70027. doi:10.1002/edm2.70027

15. Nayak SS, Kuriyakose D, Polisetty LD, Patil AA, Ameen D, Bonu R, et al. Diagnostic and prognostic value of triglyceride glucose index: a comprehensive evaluation of meta-analysis. Cardiovasc Diabetol. 2024;23(1):310. doi:10.1186/s12933-024-02392-y

16. Rauscher FG, Elze T, Francke M, Martinez-Perez ME, Li Y, Wirkner K, et al. Glucose tolerance and insulin resistance/sensitivity associate with retinal layer characteristics: the LIFE-Adult-Study. Diabetologia. 2024;67(5):928-39. doi:10.1007/s00125-024-06093-9

17. Sanchez-Pozos K, Monroy-Escutia J, Jaimes-Santoyo J, Granados-Silvestre MLA, Menjivar M, Ortiz-Lopez MG. Risk factors associated with diabetic neuropathy in Mexican patients. Cir Cir. 2021;89(2):189-99. doi:10.24875/CIRU.20000243

18. Seufert J, Freemantle N, Guja C, Haluzik M, Tournay M, Vera C, et al. Real-life effectiveness of iGlarLixi (insulin glargine 100 U/mL and lixisenatide) in people with type 2 diabetes according to prior insulin use. Diabetes Obes Metab. 2024;26(7):2988-92. doi:10.1111/dom.15609

19. Tian J, He W, Gao J, Yan L, Liang M, Zhang W, et al. Superficial Femoral Artery Calcification Is a Novel Risk Factor of Microvascular Complications in T2DM Patients. Calcif Tissue Int. 2020;106(4):355-63. doi:10.1007/s00223-019-00645-7

20. Tong XW, Zhang YT, Yu ZW, Pu SD, Li X, Xu YX, et al. Triglyceride Glucose Index is Related with the Risk of Mild Cognitive Impairment in Type 2 Diabetes. Diabetes Metab Syndr Obes. 2022;15:3577-87. doi:10.2147/DMSO.S389327

21. Wang Y, Chen H. Clinical application of cluster analysis in patients with newly diagnosed type 2 diabetes. Hormones (Athens). 2025;24(1):109-22. doi:10.1007/s42000-024-00593-4

22. Wang Y, Miao Y, Wan Q. Association Between Triglyceride-Glucose Index and Waist-To-Hip Ratio with Type 2 Diabetic Retinopathy in the Chinese Population: A Cross-Sectional Study. Endocr Res. 2024;49(4):203-12. doi:10.1080/07435800.2024.2364719

23. Xu YX, Pu SD, Zhang YT, Tong XW, Sun XT, Shan YY, et al. Insulin resistance is associated with the presence and severity of retinopathy in patients with type 2 diabetes. Clin Exp Ophthalmol. 2024;52(1):63-77. doi:10.1111/ceo.14344

24. Yang J, Zhou KF, Tao GD, Wei B, Lu YW. The predictive value of TyG and lipid ratios on the development of complications and hyperuricemia in patients with type 2 diabetes mellitus. Lipids. 2024;59(6):209-19. doi:10.1002/lipd.12411

25. Yasir M, Senthilkumar GP, Jayashree K, Ramesh Babu K, Vadivelan M, Palanivel C. Association of serum omentin-1, apelin and chemerin concentrations with the presence and severity of diabetic retinopathy in type 2 diabetes mellitus patients. Arch Physiol Biochem. 2022;128(2):313-20. doi:10.1080/13813455.2019.1680698

26. Yu L, Li B. Association Between Triglyceride-Glucose Index and Diabetic Retinopathy: A Meta-Analysis. Horm Metab Res. 2024;56(11):785-94. doi:10.1055/a-2279-7112

27. Zhou J, Zhu L, Li Y. Association between the triglyceride glucose index and diabetic retinopathy in type 2 diabetes: a meta-analysis. Front Endocrinol (Lausanne). 2023;14:1302127. doi:10.3389/fendo.2023.1302127

28. Hameed EK, Abdul-Qahar ZH, Kadium TE. The Association of Triglycerides Glucose Index with Diabetic Retinopathy in Patients with Type 2 Diabetes Mellitus. Indian Journal of Public Health Research & Development. 2019;10(11):1885-90. doi:10.5958/0976-5506.2019.03828.2

29. Chiu H, Tsai HJ, Huang JC, Wu PY, Hsu WH, Lee MY, et al. Associations between Triglyceride-Glucose Index and Micro- and Macro-angiopathies in Type 2 Diabetes Mellitus. Nutrients. 2020;12(2):328. doi:10.3390/nu12020328

30. Pang M, Wei Y, Weng X. Analysis of risk factors of type 2 diabetic retinopathy. J Xinxiang Med Univ. 2020;37(3):270-3. doi:10.7683/xxyxyxb.2020.03.017

31. Srinivasan S, Singh P, Kulothungan V, Sharma T, Raman R. Relationship between triglyceride glucose index, retinopathy and nephropathy in Type 2 diabetes. Endocrinol Diabetes Metab. 2021;4(1):e00151. doi:10.1002/edm2.151

32. Yao L, Wang X, Zhong Y, Wang Y, Wu J, Geng J, et al. The Triglyceride-Glucose Index is Associated with Diabetic Retinopathy in Chinese Patients with Type 2 Diabetes: A Hospital-Based, Nested, Case-Control Study. Diabetes Metab Syndr Obes. 2021;14:1547-55. doi:10.2147/DMSO.S294408

33. Pan Y, Zhong S, Zhou K, Tian Z, Chen F, Liu Z, et al. Association between Diabetes Complications and the Triglyceride-Glucose Index in Hospitalized Patients with Type 2 Diabetes. J Diabetes Res. 2021;2021:8757996. doi:10.1155/2021/8757996

34. Wang J, Zhang H-F, Li C-H. Triglyceride and glucose index as a predictive factor for diabetic retinopathy in Type 2 diabetic patients. International Eye Science. 2022:1385-90.

35. Li Q, Shao X, Zhou S, Cui Z, Liu H, Wang T, et al. Triglyceride-glucose index is significantly associated with the risk of hyperuricemia in patients with diabetic kidney disease. Sci Rep. 2022;12(1):19988. doi:10.1038/s41598-022-23478-1

36. Shan Y, Wang Q, Zhang Y, Tong X, Pu S, Xu Y, et al. High remnant cholesterol level is relevant to diabetic retinopathy in type 2 diabetes mellitus. Lipids Health Dis. 2022;21(1):12. doi:10.1186/s12944-021-01621-7

37. Kassab HS, Osman NA, Elrahmany SM. Assessment of Triglyceride-Glucose Index and Ratio in Patients with Type 2 Diabetes and Their Relation to Microvascular Complications. Endocr Res. 2023;48(4):94-100. doi:10.1080/07435800.2023.2245909

38. Neelam K, Aung KCY, Ang K, Tavintharan S, Sum CF, Lim SC. Association of Triglyceride Glucose Index with Prevalence and Incidence of Diabetic Retinopathy in a Singaporean Population. Clin Ophthalmol. 2023;17:445-54. doi:10.2147/OPTH.S382336

39. Shang L, Wei J, Xie Q, Li Y. Predictive value of the combination of serum TyG index, nesfatin-1, and retinol-binding protein 4 for diabetic retinopathy. International Eye Science. 2024;24(11):1802-6. doi:10.3980/j.issn.1672-5123.2024.11.20

40. Wan X, Zhang R, Abudukeranmu A, Wei W, Zhu H, Zhang L, et al. Elevated Triglyceride-Glucose Index as a Risk Stratification Marker for Diabetic Retinopathy in Type 2 Diabetes Mellitus: The Influence of Glycemic Control. Diabetes Metab Syndr Obes. 2025;18:743-59. doi:10.2147/DMSO.S503672

41. Li J, Li Y, Qi Q, Chen N, Zhang Y. Predictive value of triglyceride glucose index and systemic inflammation index for diabetic retinopathy in Type-2 diabetes. Pak J Med Sci. 2025;41(4):1072-7. doi:10.12669/pjms.41.4.11374

42. Yao Q, Liu SQ. Association of triglyceride glucose index with diabetic retinopathy in middle-aged and elderly ambulatory type 2 diabetic patients. Front Endocrinol (Lausanne). 2025;16:1442230. doi:10.3389/fendo.2025.1442230

43. Zhou Y, Lu Q, Zhang M, Yang L, Shen X. The U-Shape Relationship between Triglyceride-Glucose Index and the Risk of Diabetic Retinopathy among the US Population. J Pers Med. 2023;13(3):495. doi:10.3390/jpm13030495
